# An Online Intervention for Co-Occurring Depression and Problematic Alcohol Use in Young People: Primary Outcomes From a Randomized Controlled Trial

**DOI:** 10.2196/jmir.5178

**Published:** 2016-03-23

**Authors:** Mark Deady, Katherine L Mills, Maree Teesson, Frances Kay-Lambkin

**Affiliations:** ^1^National Health and Medical Research Council Centre for Research Excellence in Mental Health and Substance UseNational Drug and Alcohol Research CentreKensingtonAustralia; ^2^The University of NewcastlePriority Research Centre for Translational Neuroscience and Mental HealthCallaghanAustralia

**Keywords:** depression, alcohol use, alcohol abuse, problem drinking, young people at risk populations, Internet, intervention online therapy, eHealth, comorbidity

## Abstract

**Background:**

Depression and problematic alcohol use represent two of the major causes of disease burden in young adults. These conditions frequently co-occur and this is associated with increased harm and poorer outcomes than either disorder in isolation. Integrated treatments have been shown to be effective; however, there remains a significant gap between those in need of treatment and those receiving it. The increased availability of eHealth programs presents a unique opportunity to treat these conditions.

**Objective:**

This study aimed to evaluate the feasibility and preliminary efficacy of an automated Web-based self-help intervention (DEAL Project) in treating co-occurring depressive symptoms and problematic alcohol use in young people.

**Methods:**

Young people (aged 18 to 25 years) with moderate depression symptoms and drinking at hazardous levels (recruited largely via social media) were randomly allocated to the DEAL Project (n=60) or a Web-based attention-control condition (HealthWatch; n=44). The trial consisted of a 4-week intervention phase with follow-up assessment at posttreatment and at 3 and 6 months postbaseline. The primary outcomes were change in depression severity according to the Patient Health Questionnaire-9 as well as quantity and frequency of alcohol use (TOT-AL).

**Results:**

The DEAL Project was associated with statistically significant improvement in depression symptom severity (d=0.71) and reductions in alcohol use quantity (d=0.99) and frequency (d=0.76) in the short term compared to the control group. At 6-month follow-up, the improvements in the intervention group were maintained; however, the differences between the intervention and control groups were no longer statistically significant, such that between-group effects were in the small to moderate range at 6 months (depression symptoms: d=0.39; alcohol quantity: d=–0.09; alcohol frequency: d=0.24).

**Conclusions:**

Overall, the DEAL Project was associated with more rapid improvement in both depression symptoms and alcohol use outcomes in young people with these co-occurring conditions relative to an attention-control condition. However, long-term outcomes are less clear.

**Trial Registration:**

Australian New Zealand Clinical Trials Registry (ANZCTR): ACTRN12613000033741; https://www.anzctr.org.au/Trial/Registration/TrialReview.aspx?id=363461 (Archived by WebCite at http://www.webcitation.org/6fpsLEGOy)

## Introduction

Major depressive disorder and alcohol use disorders are two of the top five leading causes of years of life lost to disability in the developed world [1] with young people bearing a disproportionately large share of the burden [2]. Early intervention is imperative to averting the development of more severe, ingrained morbidity [3], yet less than 25% of affected young Australians access traditional health services in a 12-month period [4]. Comorbidity across the disorder classes is common [5] and is associated with considerable adverse outcomes [6,7]. Furthermore, young people with mental health issues rate “‘coping” as a key motive for substance use [8] with comorbid disorders often maintaining and exacerbating one another [9]. Thus, there is increasing support for integrated approaches to comorbidity treatment [10,11]. Baker and colleagues [12] have demonstrated that concurrent treatment of depression and problem drinking is more effective than treating either condition alone and more effective than general counseling.

The advantages of Web-based interventions, including flexibility, anonymity, and accessibility, appear particularly useful for individuals who are less likely to access traditional services, such as young people [13]. Web-based depression and alcohol interventions have been shown to produce effect sizes equivalent to those of traditional face-to-face therapy (0.28-0.78, 0.22-0.48) [13-21], although it has been argued that this is dependent on therapist guidance; generally, interventions with little or no therapist guidance have significantly smaller treatment effect sizes [22]. Nevertheless, guided interventions are not as cost-effective to disseminate, perhaps limiting their ability to overcome traditional barriers to treatment access, particularly among young people [23]. So far, there are no youth-focused Web-based interventions reported in the research literature for individuals experiencing both depressive and alcohol problems. Furthermore, in the general population only one computerized intervention targeting both of these disorders has been evaluated: Self-Help for Alcohol/other drug use and Depression (SHADE) [24,25]*.* Evaluations of SHADE indicate electronic forms of treatment for co-occurring disorders are viable and effective, especially when combined with brief therapist guidance. Two randomized controlled trials (RCTs) have found SHADE plus therapist guidance to be associated with equivalent outcomes to those achieved by therapist-delivered treatment, with superior results as far as reducing alcohol consumption over 3 and 12 months [24,25]. The only other study of this kind examined the use of a single session of online personalized feedback and psychoeducation provided to college students; as such, it was not specifically a youth-focused intervention [26]. The study compared alcohol feedback only, depressed mood feedback only, integrated feedback, and an assessment-only condition. At 1-month follow-up, no differences in depressed mood or alcohol use were found across the conditions; however, moderator effects were present, with the interventions being more effective than controls for those with less severe baseline symptoms.

In response to this gap in evidence-based programs for depressive and alcohol problems among young people, we developed the DEpression-ALcohol (DEAL) Project, a brief, Web-based intervention for young people aged 18 to 25 years based on the SHADE program. Because the program is a self-help intervention, it is primarily aimed at those with moderate symptomatology who may not reach diagnostic cut-offs for disorders but are, nonetheless, experiencing distress and would benefit from early intervention. Such conditions have been associated with substantial impairment [27-29], particularly in young people [30]; these conditions have been shown to escalate into full alcohol use disorder in 17.0% to 38.2% of cases within 5 years [31-33] and full major depressive disorder in 10% to 25% of patients with minor depression within 3 years [27,34].

The primary aim of this study was to evaluate the feasibility and preliminary efficacy of the DEAL Project and compare outcomes relative to an attention-control condition (HealthWatch) in a RCT. Specifically, this study aimed to determine whether (1) the DEAL Project produces significantly greater pre- to posttreatment reductions in severity of depression symptoms as well as quantity and frequency of alcohol use relative to HealthWatch and (2) changes observed from pre- to posttreatment are maintained through to 6 months postbaseline.

## Methods

### Study Design

**Figure 1:**
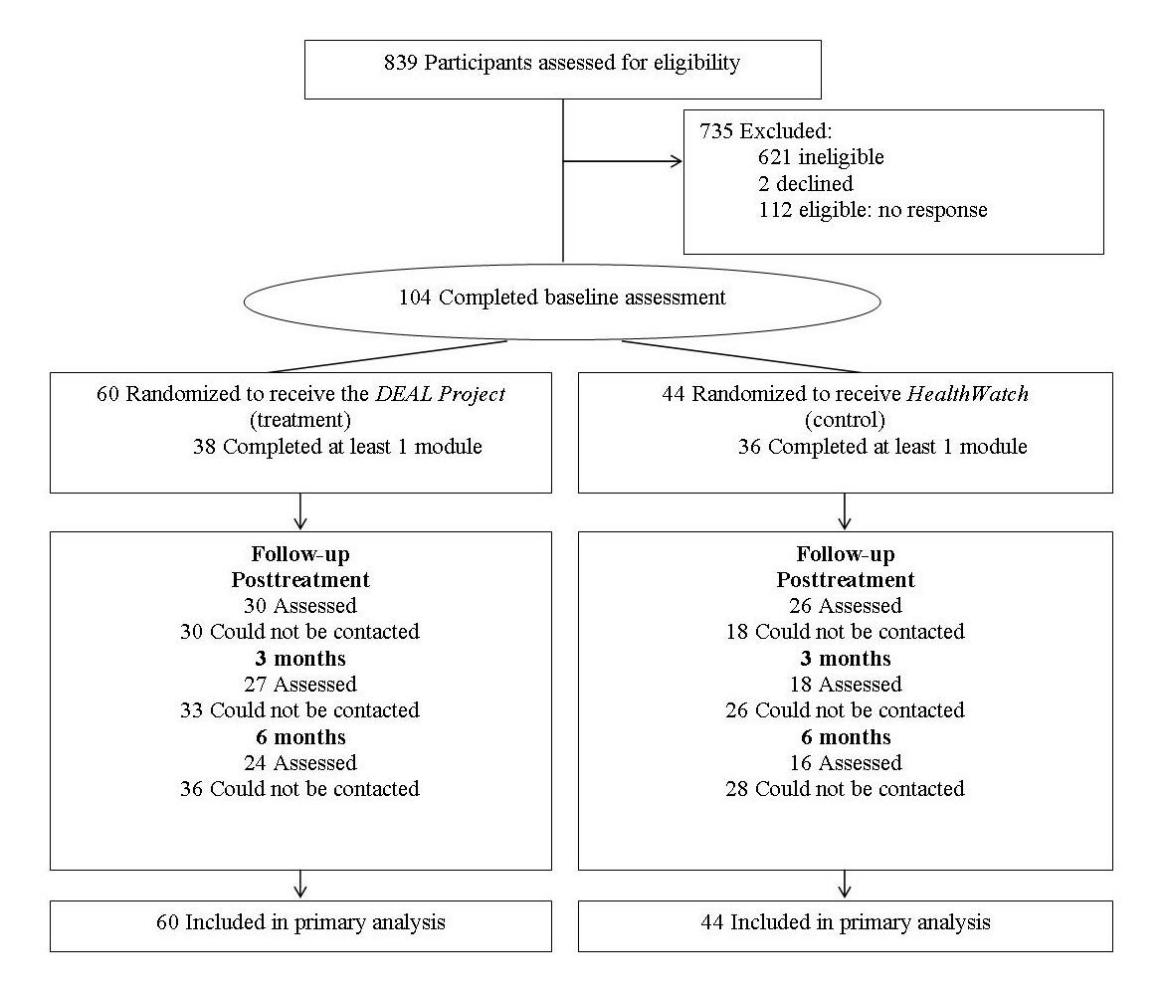
Flow of participants through study.

The study design and flow of participants is shown in Figure 1. Ethical approval was obtained from the University of New South Wales Human Research Ethics Committee and consent was obtained electronically from all participants. The study was conducted entirely online with all contact occurring via automated emails. Following online screening, eligible participants were asked to provide informed consent in order to take part and were randomized to one of two conditions delivered over four weekly sessions: (1) the DEAL Project or (2) HealthWatch. Access to the website for each of the programs was for a period of 10 weeks from the point of randomization. Randomization was automated within the online program; therefore, the trial researcher was blind to randomization. This process occurred immediately after the eligibility screener, consent form, and baseline assessment were completed. Block randomization was conducted with a 1:1 ratio; however, due to a programming error (which included test users within the blocks), a group imbalance occurred resulting in 60 participants being randomized to the DEAL Project and 44 randomized to HealthWatch. Participants then completed a baseline assessment on entry to the study and follow-up assessments at posttreatment (5 weeks), and at 3 and 6 months postbaseline.

### Recruitment

Recruitment took place between July 2013 and February 2014 using extensive media coverage across Australia, including tertiary institution flyers and street press, radio and newspaper stories, treatment services websites, and paid Facebook and Google advertisements. Individuals were eligible for the study if they (1) were aged 18 to 25 years, (2) reported current moderate depression symptomology (score of ≥7 on the Depression Anxiety Stress Scale [DASS-21-Depression]) [35], (3) were drinking at hazardous levels as measured by the Alcohol Use Disorders Identification Test (AUDIT; score of ≥8) [36], (4) had the ability to access the Internet (either in the private residence of the participant or willingness to use the public library/other suitable venue with Internet access), and (5) were Australian residents. Exclusion criteria included (1) a Psychosis Screener score ≥3 [37], (2) an inability to speak English, (3) serious risk of suicide in the past 2 weeks (serious thoughts of suicide and desire to act), and (4) daily use of cannabis/weekly use of amphetamines.

### Interventions

#### The DEAL Project

The DEAL Project [38] consists of four 1-hour modules to be completed over a 4-week period (homework is provided at the conclusion of each module and reviewed at the beginning of the subsequent module). The website tracked participants’ completion of each weekly module, with automated email reminders sent to participants’ nominated email addresses. As mentioned, the program is based on the SHADE program, which consists of evidence-based cognitive behavioral therapy and motivational interviewing. The modules were as follows:

1. Week 1: Where Are You At? Psychoeducation, assessment, goal setting, mood/activity/alcohol use monitoring (homework: mood/drinking monitoring).
2. Week 2: Getting Moving Again. Behavioral activation, decisional balance (alcohol use), behavior change, activity scheduling (homework: activity planning).
3. Week 3: Taking Charge of your Thoughts. Mood monitoring, cognitive restructuring (homework: thought monitoring).
4. Week 4: Coping with Tough Situations. Mindfulness and relaxation, problem solving, drink reduction and refusal, relapse planning and management (mindfulness/relaxation, problem-solving exercises).

#### HealthWatch

HealthWatch is a 12-module attention-control condition first developed for the Australian National University WellBeing Study [39] in which participants read information about various health concerns and complete accompanying surveys. The purpose of this condition was to control for time spent interacting with an online program. Four modules deemed to be most relevant to younger people were selected to act as the attention control in this study: environmental health, physical and mental activity, nutrition, and relationships. These were redesigned to match the DEAL Project in appearance.

### Assessment and Outcome Measures

A structured online assessment was conducted at each of the four assessment time points. Primary outcomes were (1) depressed mood as measured by the Patient Health Questionnaire-9 (PHQ-9) [40] and (2) quantity and frequency of alcohol use as measured by the TOT-AL [41]. The PHQ-9 is a reliable and valid brief measure of depression severity over the past 2 weeks [42]. The self-report measure utilizes a 4-point Likert scale ranging from 0 (not at all) to 3 (nearly every day). Total scores range from 0 to 27. Scores ≤4 are considered to represent minimal depression, scores 5-9 are considered mild, 10-14 are considered moderate, 16-19 are considered moderately severe, and scores ≥20 are considered indicative of severe depression. The TOT-AL has been found to be a reliable and valid online measure of alcohol consumption [41]. The tool uses a dropdown menu of type, brand, and size of beverages consumed each day for the past week. The TOT-AL calculates the cumulative unit content of the drinks consumed over the previous 7 days (1 unit=approximately 8 g ethanol) to generate an overall consumption score (drinks per week) and frequency score (drinking days per week).

Automatic email prompts to complete online follow-up assessments were sent to participants at baseline, posttreatment (5 weeks), and 3 and 6 months postbaseline. Reminder emails were sent if the participant did not complete an assessment within 6 days (three emails per assessment in total). On completion of assessments, participants received an AU $10 iTunes voucher as reimbursement for time.

### Data Analysis

#### Sample Size Calculation

Power analysis on the primary outcome variables was conducted using Power Analysis and Sample Size Software (PASS) [43]. Taking into account sample attrition, the sample size had 92.9% power to detect a 5-point time-averaged difference between groups (SD 6.80) on the PHQ-9 at α*<*.05 (large effect). There was 75.7% power to detect a time-averaged difference between groups of three drinks per drinking day (SD 5.89) at α*<*.05. A 5-point difference on the PHQ-9 was chosen because this was considered to be clinically significant [44]. No clinical indicators were available for the TOT-AL, but three drinks per drinking day was considered substantial enough to be significant at a public health level.

### Statistical Analysis

Data were analyzed using PASW Statistics 18 for Windows (release 18.0.0) [45]. Baseline differences between groups were examined using chi-square (using Yates continuity correction in 2×2 tables to avoid overestimation) and independent-samples *t* tests.

Analysis of outcomes was undertaken based on intention to treat using a series of generalized estimating equations (GEE) [46]. Baseline scores were modeled and controlled for in the GEE analysis. Linear and negative binomial (with log link) GEE were used to examine outcomes with normal and count distributions, respectively. An exchangeable correlation structure was used for all models. An interaction term for the intervention variable and time (group × time) was included in each model to test for differential change over time. When this interaction was nonsignificant (*P*≥.05), it was removed from the model and the analysis was rerun. Alpha was set at .05 and a least significant difference method was used for pairwise comparisons. Results are reported as the unstandardized mean difference (beta) with Wald-type 95% confidence interval (95% CI) and chi-square for linear models and the risk ratio (RR) with 95% CI for negative binomial models. Furthermore, standardized effect sizes (Cohen’s *d*) were calculated for the primary outcomes [47].

#### Sample Retention and Missing Data

The follow-up rates at posttreatment and at 3 and 6 months postbaseline were 53.8% (56/104), 43.3% (45/104), and 38.5% (40/104), respectively. Those who completed follow-up assessments were consistently more likely to have completed a session (χ^2^_1_=15.3, *P*<.001), seen a psychologist in past year at baseline (χ^2^_1_=5.3, *P=*.02), and were less likely to be a smoker (χ^2^_1_=5.1, *P=*.02). Importantly, attrition was not related to treatment allocation or any of the outcome variables of interest.

Missing data analysis revealed 35.79% (2866/8008) missing data across all follow-up assessments. According to the results of Little’s missing completely at random test [48], the data could be considered to be missing completely at random (χ^2^_711_=652.5, *P=*.94).

## Results

### Participant Characteristics

The sample consisted of 104 participants (female: 59.6%, 62/104) with a mean age of 21.74 (SD 2.22) years. The mean PHQ-9 score was 16.32 (SD 5.00) and the mean AUDIT score was 17.39 (SD 6.42). Drinking quantities and frequencies were positively skewed with a median of 15.20 standard alcoholic drinks per week (range 0-97) consumed over a median of 2.00 drinking days per week (range 0-7). There were no statistically significant differences between the groups on baseline characteristics (Table 1).

**Table 1:** Participant characteristics by group.

Demographics		DEAL Project treatment (n=60)	HealthWatch control (n=44)	Total (N=104)	*t*_102_	χ^2^ (*df*)	*P*
Age (years), mean (SD)		21.85 (2.32)	21.59 (2.08)	21.74 (2.22)	0.59		.56
Sex (female), n (%)		36 (60)	26 (59)	62 (60)		0.0 (1)	>.99
Rural, n (%)		5 (8)	8 (18)	13 (13)		1.4 (1)	.23
Completed secondary school, n (%)		52 (87)	33 (75)	85 (82)		0.2 (1)	.21
**Postschool qualifications, n (%)**						3.7 (2)	.15
	No postschool	11 (18)	15 (34)	26 (25.0)			
	TAFE student/graduate	12 (20)	9 (20)	21 (20)			
	University student/graduate	37 (68)	20 (45)	57 (55)			
**Employment, n (%)**						1.8 (2)	.40
	Unemployed	7 (12)	4 (9)	11 (11)			
	Full/part time employment	42 (70)	27 (61)	69 (66)			
	Student	11 (18)	13 (30)	24 (23)			
**Mental health treatment in past year, n (%)**							
	GP	33 (55)	28 (64)	61 (59)		0.47 (1)	.49
	Psychologist	19 (32)	14 (32)	33 (32)		3.19 (1)	.07
	Psychiatrist	21 (35)	24 (55)	45 (43)		0.00 (1)	>.99
	Other mental health worker	5 (8)	10 (23)	15 (14)		3.17 (1)	.08
	Alcohol/drug worker	1 (2)	5 (11)	6 (6)		2.79 (1)	.09
	Medication, n (%)	17 (28)	14 (31.8)	31 (30)		0.03 (1)	.87
Doubtful about computer therapy, n (%)		29 (48)	22 (50)	51 (49)		0.00 (1)	>.99
Borderline symptoms (MSI-BPD), mean (SD)		5.80 (2.41)	6.50 (2.02)	6.10 (2.27)	0.59		.56
**Depression**							
	Likely MDD diagnosis (PHQ-9), n (%)	35 (58)	21 (48)	56 (54)		0.76 (1)	.38
	Likely other depressive diagnosis, n (%)	8 (13)	5 (11)	13 (13)		0.00 (1)	>.99
**Alcohol in past 12 months**							
	≥1 alcohol abuse criteria met, n (%)	39 (65)	29 (66)	68 (66)		0.00 (1)	>.99
	≥3 alcohol dependence criteria met, n (%)	50 (83)	35 (80)	85 (82)		0.06 (1)	.81
AUDIT, mean (SD)		17.02 (6.19)	17.89 (6.76)	17.38 (6.42)	–0.68		.50
Smoker, n (%)		17 (28)	17 (39)	34 (33)		0.80 (1)	.37
**Drug use in past month, n (%)**							
	Cannabis	16 (27)	9 (20)	25 (24)		0.25 (1)	.62
	Stimulants	7 (12)	7 (16)	14 (13)		0.11 (1)	.74
	Other illicit drugs	3 (5)	2 (5)	5 (5)		0.00 (1)	>.99

#### Treatment Retention

Compared to those in the control group, those in the treatment group attended fewer sessions (*t*_102_=–3.14, *P=*.002). The treatment group completed a mean of 1.50 sessions (SD 1.53), whereas the control group fully completed mean 2.50 sessions (SD 1.69). Overall, 68.3% (71/104) of the sample completed at least one module (treatment: 60.0%, 36/60; control: 79.5%, 21/44). This figure did not statistically differ significantly between groups (χ^2^_1_=3.4, *P=*.07). In both groups, missing data at follow-up was associated with fewer modules completed (posttreatment: χ^2^_4_=263.7, *P*<.001; 3 month: χ^2^_4_=82.5, *P*<.001; 6 month: χ^2^_4_=102.6, *P*<.001).

#### Service Use

At each time point, participants reported their use of a range of services for mental health treatment (medication, psychologist, alcohol and drug worker, psychiatrist, general practitioner, other health professional). Although those in the control condition were consistently more likely to use services for mental health problems over the 6-month follow-up, there was no difference in service use over the course of the trial (χ^2^_1_=3.6, *P=*.55).

### Treatment Outcomes

#### Primary Depression Outcomes

There were no statistically significant differences between the groups on PHQ-9 scores at baseline (see Table 2). There was a statistically significant group × time interaction in relation to depression symptom severity (χ^2^_3_=11.5, *P=*.009), indicating that the treatment and control groups differed on PHQ-9 scores over time. As shown in Table 2, the treatment group demonstrated a statistically significant reduction in symptom severity from baseline to posttreatment follow-up (beta=–5.94, 95% CI –8.18 to –3.70; *P*<.001), representing a large effect (*d*=1.09). The change in control group PHQ-9 scores over this time was small (*d*=0.18) and not statistically significant (beta=–1.43, 95% CI –3.46 to 0.60; *P*=.17). Overall, the degree of improvement in depression symptom severity between baseline and posttreatment follow-up was 4.51 points greater in the treatment group compared to the control group and the treatment group reported significantly better depression scores relative to control at posttreatment follow-up (beta=–3.89, 95% CI –7.09 to –0.68; *d*=0.71).

The reduction in severity of depression observed for the treatment group persisted from posttreatment to 3-month follow-up (beta=0.01, 95%CI –2.52 to 2.53; * P* <.99) and from the 3- to 6-month follow-ups (beta= –1.59, 95% CI –1.38 to 4.57; *P*=.29; i.e, no statistically significant change). The control group demonstrated a statistically significant reduction in depression symptoms between posttreatment and 3-month follow-up (beta = –2.78, 95% CI –5.33 to 0.23; *P*=.03) that persisted to the 6-month follow-up (beta= –0.61, 95% CI –2.83 to 1.60; *P*=.59).

There was no statistically significant difference in depression scores between groups at either the 3- (beta =–1.10, 95% CI –5.10 to 2.90; *P*=.59; *d*=0.15) or 6-month follow-ups (beta=–2.08, 95% CI –6.45 to 2.29; *P*=.35; *d*=0.39). The within-group effect between baseline and 3-month follow-up for the treatment group was *d*=0.96 and *d*=0.67 for the control group. The within-group effect between baseline and 6-month follow-up was *d*=1.42 for the treatment group and *d*=0.78 for the control group.

##### Primary Alcohol Outcomes

###### Drinks Per Week

There was no difference at baseline between the groups for alcohol use quantity as measured by the TOT-AL (see Table 3). There was a statistically significant group × time interaction in relation to number of standard drinks per week (χ^2^_3_=9.3, *P=*.03). As shown in Table 3, the treatment group demonstrated a significant reduction in drinks per week from baseline to posttreatment follow-up (RR=0.46, 95% CI 0.32-0.65; *P*<.001) representing a large effect (*d*=1.07). The change in drinks per week in the control group over this time was small (*d*=0.03) and not statistically significant (RR=0.97, 95% CI 0.67-1.41; *P*=.88). Overall, the treatment group reported a two-fold greater reduction in standard drinks consumed per week between baseline and posttreatment follow-up compared to the control group (RR=2.13, 95% CI 1.28-3.54; *P*=.02). Consequently, the treatment group reported statistically significantly fewer drinks per week relative to control at posttreatment follow-up (RR=0.62, 95% CI 0.39-1.00; *P*=.05).

**Table 2:** Unadjusted comparisons between conditions on Patient Health Questionnaire-9.

Time point	DEAL Project			HealthWatch			Between-group difference			
	Mean (95% CI)	Change from t0 (95% CI)	*P*	Mean (95% CI)	Change from t0 (95% CI)	*P*	Mean (95% CI)	*P*	Change from t0 (95% CI)	*P*
t0	16.58 (15.42, 17.75)	—		15.95 (14.36, 17.54)	—		0.63 (–1.34, 2.60)		—	.53
t1	10.64 (8.31, 12.97)	–5.94 (–8.18, –3.70)	<.001	14.53 (12.33, 16.73)	–1.43 (–3.46, 0.60)	.17	–3.89 (–7.09, –0.68)	.02	4.51 (1.49, 7.54)	.003
t2	10.65 (7.99, 13.31)	–5.93 (–8.53, –3.37)	<.001	11.75 (8.76, 14.74)	–4.21 (–7.27, –1.15)	.01	–1.10 (–5.10, 2.90)	.59	–1.73 (–5.74, 2.29)	.40
t3	9.05 (6.21, 11.90)	–7.53 (–10.51, –4.55)	<.001	11.14 (7.82, 14.45)	–4.82 (–8.28, 1.36)	.01	–2.08 (–6.45, 2.26)	.35	–2.71 (–7.28, 1.86)	.24

**Table 3:** Unadjusted comparisons between conditions on TOT-AL.

Time point		DEAL Project			HealthWatch			Between-group differences, RR (95% CI)			
		Mean (95% CI)	RR^a^ (95% CI)	*P*	Mean (95% CI)	RR^a^ (95% CI)	*P*	At each time point	*P*	From t0	*P*
**Drinks per week**											
	t0	25.65 (19.52-33.71)	—		19.43 (14.02-26.93)	—		1.32 (1.16-2.02)		—	
	t1	11.72 (8.11-16.93)	0.46 (0.32-0.65)	<.001	18.89 (14.00-25.52)	0.97 (0.67-1.41)	.88	0.62 (0.39-1.00)	.05	2.13 (1.28-3.54)	.02
	t2	9.79 (4.66-20.54)	0.38 (0.19-0.76)	.006	12.96 (7.65-21.96)	0.67 (0.37-1.22)	.19	0.76 (0.30-1.88)	.55	1.75 (0.70-4.73)	.23
	t3	15.81 (9.89-25.27)	0.62 (0.41-0.93)	.02	15.97 (9.87-25.84)	0.82 (0.47-1.42)	.48	0.99 (0.51-1.94)	.98	1.33 (0.67-2.65)	.41
**Drinking days per week**											
	t0	3.00 (2.49-3.60)	—		2.64 (2.05-3.41)	—		1.13 (0.83-1.55)	.43	—	
	t1	1.56 (1.18-2.07)	0.52 (0.41-0.67)	<.001	2.48 (1.89-3.25)	0.93 (0.69-1.26)	.67	0.63 (0.43-0.93)	.02	1.79 (1.22-2.64)	.003
	t2	1.59 (1.07-2.34)	0.53 (0.37-0.76)	.001	1.90 (1.15-3.13)	0.72 (0.42-1.24)	.23	0.84 (0.44-1.58)	.58	1.35 (0.70-2.61)	.36
	t3	2.07 (1.46-3.13)	0.69 (0.50-0.96)	.03	2.67 (1.71-4.15)	1.01 (0.64-1.59)	.97	0.78 (0.44-1.37)	.38	1.46 (0.83-2.55)	.19

^a^ From t0.

The reduction observed for the treatment group persisted from posttreatment to 3-month follow-up (RR=0.84, 95% CI 0.46-1.51; *P*=.55). Between the 3- and 6-month follow-ups, the number of drinks per week in the treatment group increased (RR=1.62, 95% CI 0.96-1.90; *P*=.04); however, at 6-month follow-up the number of drinks per week was still significantly lower than baseline (RR=0.62, 95% CI 0.41-0.93; *P*=.02). No statistically significant change in the number of drinks per week was found for the control group between posttreatment and 3-month follow-up (RR=0.69, 95% CI 0.44-1.07; *P*=.10) or between the 3- and 6-month follow-ups (RR=1.23, 95% CI 0.61-2.50; *P*=.56). Similarly, compared to baseline, the number of drinks per week in the control group was no different at the 3- (RR=0.67, 95% CI 0.37-1.22; *P*=.19) or 6-month follow-ups (RR=0.82, 95% CI 0.47-1.42; *P*=.48).

There was no statistically significant difference in the number of drinks per week between the treatment and control groups at either the 3- (RR=0.76, 95% CI 0.30-1.88; *d*=0.13; *P*=.55) or 6-month follow-ups (RR=0.99, 95% CI 0.51-1.94; *d* =–0.09; *P*=.99). The within-group effect between baseline and 3-month follow-up for the treatment group was *d*=0.76 and *d*=0.54 for the control group. The within-group effect between baseline and 6-month follow-up was *d*=0.38 for the treatment group and *d*=0.24 for the control group.

###### Drinking Days Per Week

There were no statistically significant differences between the groups for alcohol use frequency as measured by the TOT-AL (see Table 3). There was a statistically significant group × time interaction in relation to number of drinking days per week (χ^2^_3_=9.6, *P=*.02). As shown in Table 3, the treatment group demonstrated a statistically significant reduction in drinking days per week from baseline to posttreatment follow-up (RR=0.52, 95% CI 0.41-0.67; *P*<.001), representing a large effect (*d*=1.06). The change in weekly drinking days in the control group over this time was small (*d*=0.10) and not statistically significant (RR=0.93, 95% CI 0.69-1.26; *P*=.67). Compared to the control group, the treatment group reported a 79% greater reduction in drinking days (RR=1.79, 95% CI 1.22-2.64; *P=*.003). The treatment group also reported significantly fewer drinking days per week relative to control at posttreatment follow-up (RR=0.63, 95% CI 0.43-0.93; *d=*0.76; *P*=.02).

The reduction observed for the treatment group persisted from posttreatment to 3-month follow-up (RR=1.01, 95% CI 0.70-1.47; *P*=.94) and from 3- to 6-month follow-ups (RR=1.31, 95% CI 0.93-1.84). No statistically significant change was observed for the number of drinking days per week in the control group between posttreatment and 3-month follow-up (RR=0.77, 95% CI 0.49-1.20; *P*=.25) or between 3- and 6-month follow-ups (RR=0.71, 95% CI 0.32-1.57; *P*=.40). Similarly, compared to baseline, the number of drinking days per week in the control group was no different at 3- (RR=0.72, 95% CI 0.42-1.23; *P*=.23) or 6-month follow-ups (RR=1.01, 95% CI 0.64-1.59; *P*=.97).

There was no statistically significant difference in the number of drinking days per week between groups at either the 3- (RR=0.84, 95% CI 0.44-1.58; *P*=.58; *d*=0.22) or 6-month follow-ups (RR=0.78, 95% CI 0.44-1.37; *P*=.38; *d=*0.24). The within-group effect between baseline and 3-month follow-up for the treatment group was *d*=0.89 and *d*=0.45 for the control group. The within-group effect between baseline and 6-month follow-up was *d*=0.42 for the treatment group and *d*=0.04 for the control group.

### Discussion

This RCT evaluated the feasibility and preliminary efficacy of the DEAL Project*,* a Web-based program that aims to reduce depression and alcohol use in 18- to 25-year-olds. The program demonstrated statistically significant greater reductions in depression and alcohol use compared to a control group at posttreatment. Furthermore, the positive outcomes observed among those randomized to the DEAL Project were maintained at 3- and 6-month follow-ups. However, between-group differences at these later time points disappeared because of statistically nonsignificant shifts in both control and treatment groups. There is evidence to suggest that in brief intervention trials, assessment alone may result in improved outcomes either as a consequence of assessment on subsequent self-report (known as the Hawthorne effect) [49] or as a catalyst to mobilize individuals into actual behavioral change [25]. As such, there is the potential that those in the HealthWatch condition may have derived benefit from not only the assessments, but also the thought involved in completing the surveys. Alternately, because this was a sample that sought out this treatment, the control condition may have been intensive enough for some change to be observed when combined with participant motivation for change. Participants may have also accessed other treatments. However, this was not borne out in our data on service utilization over the follow-up period. Treatment deterioration effects may also have led to this lack of between-group differences at 6 months. Finally, natural recovery cannot be disregarded as an alternative explanation for disappearance of differences between the two groups. Nevertheless, it would appear that the DEAL Project was associated with more rapid improvement in depression and alcohol outcomes compared with control.

Overall, mean PHQ-9 depression scores in the treatment group dropped from the “moderately severe” range to just outside the range for “mild depression” at 6 months. This was a clinically significant change [44]. At baseline, the treatment group were drinking, on average, 3 days per week and consuming more than 25 drinks per week. At 3-month follow-up, drinking occasions had halved and participants were drinking just over nine drinks per week. Although this reduced figure is still considered above the recommended range for short-term harm [50], it has potentially large public health implications (especially considering the automated nature of the program), including a reduction in risk of harm to the individual and the societal costs associated with heavy alcohol use, including violence, hospital and emergency department visits, road safety and drunk driving, and lost productivity [51]. The posttreatment effect sizes observed in this study were considerably stronger than previous research; however, at 3- and 6-month follow-ups, effect sizes looked similar to the small and moderate effects found in previous Internet-based trials for single disorder interventions [14,16,19,52,53]. Similarly, the overall effects of the DEAL Project program at 3- and 6-month follow-ups were similar to those of the SHADE program trial [24]. The DEAL Project is briefer, unguided, and delivered completely online, which is likely to increase cost-effectiveness and accessibility for youth.

Despite these various strengths, this study is not without its limitations. Although there was significant interest at the recruitment level (with more than 900 individuals beginning—and 839 completing—the screener over a 6-month period), the recruitment rate was low. However, this was unsurprising considering this was an opportunistic sample (not treatment-seeking). Consequently, on average, individuals randomized to the DEAL Project fully completed less than half of the four sessions offered to them. Of those who completed one session, approximately one-third went on to fully complete the program. This issue with adherence is unsurprising given the unguided nature of the program [54,55]. Despite considerable efforts in the program development stage to optimize adherence [56], this raises some questions around program acceptability and feasibility. Interestingly, the mean number of DEAL Project modules completed was equivalent to the number of modules completed by the younger participants in the previous SHADE trial [57], suggesting that adherence may be a generalized issue for this demographic. The DEAL Project’s brief structure allows for exposure to more key strategies before dropout. Although only one-third of the treatment group completed at least half of the program, module completion refers to full completion and does not account for participants sampling from different modules in a nonlinear way. Furthermore, the reasons for selective attrition are difficult to interpret because they may reflect the contradictory possibilities of dropout due to dissatisfaction versus dropout due to a sense that the individual feels their needs have been met [58]. Further research is needed to examine methods to improve engagement and retention within online programs. Nevertheless, despite the lack of adherence the positive results are encouraging for the utility of brief interventions. Similarly, although not dissimilar to previous studies without therapist/administrative guidance of participants [59,60], the follow-up rates were low. This may limit the generalizability and conclusions that may be drawn from this study. Telephone or face-to-face contact during online trials has been shown to increase adherence; however, this reduces real-world applicability [61]. Nevertheless, the GEE analysis used is robust to this level of loss to follow-up [62] and missingness was found to be at random.

Due to difficulties diagnosing disorders online, the sample was nondiagnostic. Nevertheless, the mean baseline PHQ-9 score was in the moderately severe range and the mean baseline AUDIT score indicated high-risk/harmful levels of use. According to the PHQ-9, more than half the sample had a likely depression diagnosis, whereas two-thirds endorsed at least one alcohol abuse diagnostic criterion and three-quarters endorsed more than two dependence diagnostic criteria. Thus, this was not a clinically insignificant sample. Furthermore, subthreshold conditions have been associated with comparable negative outcomes to full-disorder syndromes, especially in younger populations [63,64], and provide an opportunity for early intervention.

Additionally, this Australian sample may not generalize internationally. The programming error in randomization is a possible study limitation. Nevertheless, few differences were found between the groups at baseline. Finally, as with any study of this kind, there is the potential for self-report bias; evidence suggests self-report provides useful and accurate estimates when conditions are designed to maximize response accuracy [65]. Studies have shown that self-reports of alcohol use correlate with behavioral observations [66]. Furthermore, the anonymity provided by online assessment is likely to be more accurate than other forms of self-report [67].

Overall, the DEAL Project was associated with significant improvements in both depression symptoms and alcohol use among young people with these co-occurring conditions relative to control at posttreatment. However, although within-group improvements were maintained over the 6-month follow-up period, the significant between-group differences were no longer present at long-term follow-up. This study adds useful evidence to both the eHealth and comorbidity treatment fields. Further studies are required to better understand these long-term outcomes and address the program adherence and trial attrition issues that were present in this study.

## Abbreviations

DASS: Depression Anxiety Stress Scale
AUDIT: Alcohol Use Disorders Identification Test
GEE: generalized estimating equations
MSI-BPD: McLean Screening Instrument for Borderline Personality Disorder
PHQ-9: Patient Health Questionnaire
RCT: randomized controlled trial
RR: risk ratio
SHADE: Self-Help for Alcohol and other drug use and DEpression
